# Bryophytes of Santa Maria Island (Azores, Portugal): an updated inventory

**DOI:** 10.3897/BDJ.14.e189834

**Published:** 2026-05-11

**Authors:** Leila N. Morgado, Clara Polaino-Martín, Silvia Poponessi, Gabriela M. Silveira, Mariana A. Sousa, Paulo A. V. Borges, Rosalina Gabriel

**Affiliations:** 1 University of Azores, CE3C—Centre for Ecology, Evolution and Environmental Changes, Azorean Biodiversity Group, CHANGE —Global Change and Sustainability Institute, School of Agricultural and Environmental Sciences, Rua Capitão João d’Ávila, Pico da Urze, 9700-042, Angra do Heroísmo, Azores, Portugal University of Azores, CE3C—Centre for Ecology, Evolution and Environmental Changes, Azorean Biodiversity Group, CHANGE —Global Change and Sustainability Institute, School of Agricultural and Environmental Sciences, Rua Capitão João d’Ávila, Pico da Urze, 9700-042 Angra do Heroísmo, Azores Portugal; 2 Associação “Os Montanheiros”, Angra do Heroísmo, Portugal Associação “Os Montanheiros” Angra do Heroísmo Portugal; 3 University of Cagliari, Cagliari, Italy University of Cagliari Cagliari Italy; 4 University of the Azores, Gaspar Frutuoso Foundation, Rua Capitão João d´Ávila, Pico da Urze, 9700-042, Angra do Heroísmo, Azores, Portugal University of the Azores, Gaspar Frutuoso Foundation, Rua Capitão João d´Ávila, Pico da Urze, 9700-042 Angra do Heroísmo, Azores Portugal

**Keywords:** Azores, bryoflora, diversity, elevational gradient, GIMS protocol, hornworts, liverworts, Macaronesia, mosses, MOVECLIM-AZO, opportunistic approach sampling, natural park, Santa Maria Island, substrates, Wallacean shortfall.

## Abstract

**Background:**

A bryophyte survey was conducted on Santa Maria Island in 2019, taking advantage of the MOVECLIM-AZO project expedition to the Island, while adding collections in different sites. The standard collection follows the Global Island Monitoring Scheme (GIMS) protocol for bryophytes across three altitudinal levels (16, 200 and 400 m a.s.l.) and substrates, while the other specimens were collected opportunistically to improve the known dataset of bryophyte occurrences in Santa Maria. Opportunistic sampling was carried out guided by taxonomic expertise, while visiting different locations between 31 and 464 m a.s.l. and exploring diverse substrates. The survey resulted in the collection of 85 species and 810 specimens (occurrence records), obtained from 264 events. This research presents a dataset that expands current knowledge about bryophyte diversity on Santa Maria Island and supports future studies.

**New information:**

A total of 85 species were recorded on Santa Maria Island, based on 264 event records and 810 occurrence records, including 25 species newly recorded for the Island. Of these, two taxa are Azorean endemics (*Rhynchostegiella azorica* Hedenäs & Vanderp. and Leptoscyphus porphyrius subsp. azoricus (H.Buch & Perss.) Vanderp. & Heinrichs), one species (*Calypogeia azorica* Bischl.) is recognised as a Macaronesian endemic, three species (*Lejeunea hibernica* Bischl., H.A.Mill. & Bonner ex Grolle, *Radula aquilegia* (Hook.f. & Taylor) Gottsche, Lindenb. & Nees and *Radula holtii* Spruce) are European endemics, 18 species are classified as native and one species (*Campylopus introflexus* (Hedw.) Brid.) is classified as introduced.

## Introduction

Since 2012, considerable efforts have been made to update knowledge of the bryoflora in the Azores, particularly through the MOVECLIM project, "Montane Vegetation as Listening Posts for Climate Change" (Ah-Peng et al. 2012, Gabriel et al. 2014, Borges et al. 2018 and several publications in the Azores Biota (2020)). This initiative aimed to characterise the highly diverse, yet poorly documented bryophyte communities across several archipelagos, including the Azores, the Canary Islands and the Mascarene Islands, by applying standardied sampling across all available substrates along elevational gradients. In the Azores, seven islands were surveyed using a modified version of the MOVECLIM protocol (Ah-Peng et al. 2012, Gabriel et al. 2014, Borges et al. 2018), with Pico and Terceira Islands being surveyed in 2012 (Coelho et al. 2021, Gabriel et al. 2024a, Gabriel et al. 2024b), Flores (Gabriel et al. 2025a, Gabriel et al. 2025b) and São Miguel in 2013, São Jorge (Silveira and Gabriel 2026) in 2014, Faial in 2014/2015 and Santa Maria in 2019 (Morgado et al. 2026).

Before this study, 206 bryophyte species had been recorded on Santa Maria Island: 131 mosses, 72 liverworts and three hornworts, which is a small proportion of the more than 500 species present in the Azores (Gabriel et al. 2010, PBA 2026). This may be related to the small size and low elevation of the Island (Forjaz 2004), to the prevalence of modified habitats (Borges et al. 2020), but most likely to a deficit of sampling. This Island is one of the least studied compared with the islands of Terceira (Gabriel et al. 2024a) and Pico (Gabriel et al. 2024b), as only a few bryologists have collected specimens there (Suppl. material 1). Particularly noteworthy are the works of Trelease (1897), Allorge and Allorge (1950), Allorge and Allorge (1952), Crundwell et al. (1994), Sjögren (1996) and Soares (2003).

Continued efforts in bryophyte inventorisation, monitoring and habitat protection are essential not only to understand and safeguard these cryptogamic components of Santa Maria Island’s biodiversity, but also to help reduce the Wallacean shortfall (Cardoso et al. 2011) by refining knowledge on species distributions, range limits and patterns of rarity in Macaronesian bryophytes.

## General description

### Purpose

This study aims to: (i) update the inventory of the bryophyte flora of Santa Maria Island across different elevation levels and substrates, by documenting species occurrences and distributional data derived from both stratified and opportunistic sampling conducted in 2019; (ii) compile and systematise the relevant literature on bryophytes in Santa Maria; and (iii) expand the bryophyte collection of the Herbarium of the University of the Azores (AZU – Bryophyte Section).

### Additional information

The implementation of the Darwin Core Database was performed under the scope of the ongoing project BioMonI – Biodiversity monitoring of Island ecosystems project under the 2022-2023 BiodivMon joint call, co-funded by the European Commission (GA No. 101052342).

## Project description

### Title

The MOVECLIM – AZORES project: Bryophytes from Santa Maria Island (2019)

### Personnel

The MOVECLIM project was originally initiated by the research team led by Claudine Ah-Peng with the aim of studying and promoting tropical bryophytes and ferns as bioindicators of climate change. The bryoflora inventory on Santa Maria Island was coordinated by Rosalina Gabriel, with fieldwork carried out in collaboration with Clara Polaino-Martín. Most species identifications were made by Silvia Poponessi, with the contributions of Gabriela M. Silveira and Mariana A. Sousa, under the supervision of Rosalina Gabriel. Species of the genus *Frullania* were reviewed by Leila N. Morgado.

### Study area description

Located in the North Atlantic Ocean (36°55′–39°43′ N and 25°00′–31°15′ W), the Azores Archipelago comprises nine volcanic islands, organised into three geographical groups: the Western Group (Flores; Corvo), the Central Group (Terceira; Pico; São Jorge; Faial; Graciosa) and the Eastern Group (São Miguel; Santa Maria). The Archipelago is located approximately 1500 km from the western European coast and about 3900 km from the North American coast (Forjaz 2004).

Santa Maria is the oldest island of the Azores Archipelago (~ 6 Ma) (Ramalho et al. 2017), having emerged above sea level during the Late Miocene (Sibrant et al. 2015). The Island covers an area of 95.9 km² and its highest point is Pico Alto, which reaches an elevation of 587 m (Forjaz 2004). The vegetation of Santa Maria Island reflects the influence of biogeography, climate and long-term human influence. Historically, the Island supported extensive native forest formations characterised by depauperate Laurisilva and other endemic woodland types (e.g. *Juniperus* forests); however, these have been considerably reduced due to land-use change and agricultural expansion following human settlement. Currently, remnant patches of native vegetation persist, particularly in areas such as Pico Alto, where *Laurus azorica* (Seub.) Franco, *Picconia azorica* (Tutin) Knobl. and *Morella faya* (Aiton) Wilbur still occur (Elias et al. 2016, Elias 2025), although no *Juniperus brevifolia* is currently found on the Island.

### Design description

Bryophyte data were collected during fieldwork from 16–20 September 2019, following the GIMS sampling protocol (Borges et al. 2018), which is based on the BRYOLAT methodology (Ah-Peng et al. 2012) and incorporates a range of environmental variables (Gabriel et al. 2014). In addition, a non-systematic, opportunistic *ad hoc* approach was employed to collect bryophytes from various microhabitats according to the researchers’ experience.

In the GIMS systematic sampling, surveys were conducted along an altitudinal gradient from 16 to 400 m a.s.l., at 200 m intervals. At each site, two 10 m × 10 m plots were established in vegetation of uniform structural characteristics and subdivided into 25 quadrats (2 m × 2 m), from which three were randomly selected. In each selected quadrat, three microplots (5 cm × 10 cm) were sampled across up to six substrate types, when present: rock, soil, organic matter, decaying wood, tree bark at three vertical strata and leaves or fronds. In non-systematic sampling, observations and collections of bryophytes were made in different microhabitats and substrates between 31 and 464 m a.s.l. All specimens were manually collected into paper bags using a knife or forceps and labelled *in situ* with locality data and environmental information.

In the laboratory, specimens were identified to the lowest possible taxonomic level and both cover (estimated using the Braun-Blanquet scale) and sociability were recorded.

### Funding

This study was originally funded by the ERANET BIOME MOVECLIM project "Montane vegetation as listening posts for climate change" (Regional Government of the Azores, grant M2.1.2/F/04/2011/NET). This research was also funded by *Fundação para a Ciência e Tecnologia*, through the strategic funding of the Centre for Ecology, Evolution and Environmental Changes (CE3C; UID/00329/2025; DOI: 10.54499/UID/00329/2025), by *Direção Regional da Ciência, Inovação e Desenvolvimento* (DRCID), under the PROSCIENTIA Incentive System (M1.1. A/FUNC.UI&D/021/2025) and by the ERA-NET‑BIOME research framework through Project BioMonI (FCT-NETBIOME 0003/2011).

## Sampling methods

### Study extent

The field study was carried out in accordance with the sampling protocols (see "Design description"), adapted to the conditions and knowledge of the flora of Santa Maria Island. Most sampling points are located within the Natural Park of Santa Maria Island, established by Regional Legislative Decree No. 47/2008/A, which aims to protect the Island’s biodiversity and unique landscapes through a network of terrestrial and marine protected areas. Notable areas within the Park include the protected landscapes of Barreiro da Faneca, Baía de São Lourenço and Baía da Maia, as well as the natural monument of Pedreira do Campo (DRAAC 2020). The Island’s vegetation is currently restricted to three main forest types: the submontane Laurisilva, the basal Laurisilva and the coastal woodland (Elias et al. 2016).

### Sampling description

During systematic sampling, each quadrat was inspected for the presence of bryophyte colonised substrates, which were subsequently collected.

Non-systematic sampling consisted of opportunistic collections aimed at obtaining bryophytes from different microhabitats and substrate (rupicolous, terricolous, rupicolous–terricolous, lignicolous, epiphytic and epiphyllous). The substrate category “rupicolous–terricolous” was recorded when a specimen was growing on both substrates, meaning that it was attached to rock and soil simultaneously.

Specimens were collected into paper bags with a knife or tweezers. Each sample was labelled with the location name, altitude, plot, square, substrate type and replicate number.

**Figure 1.**
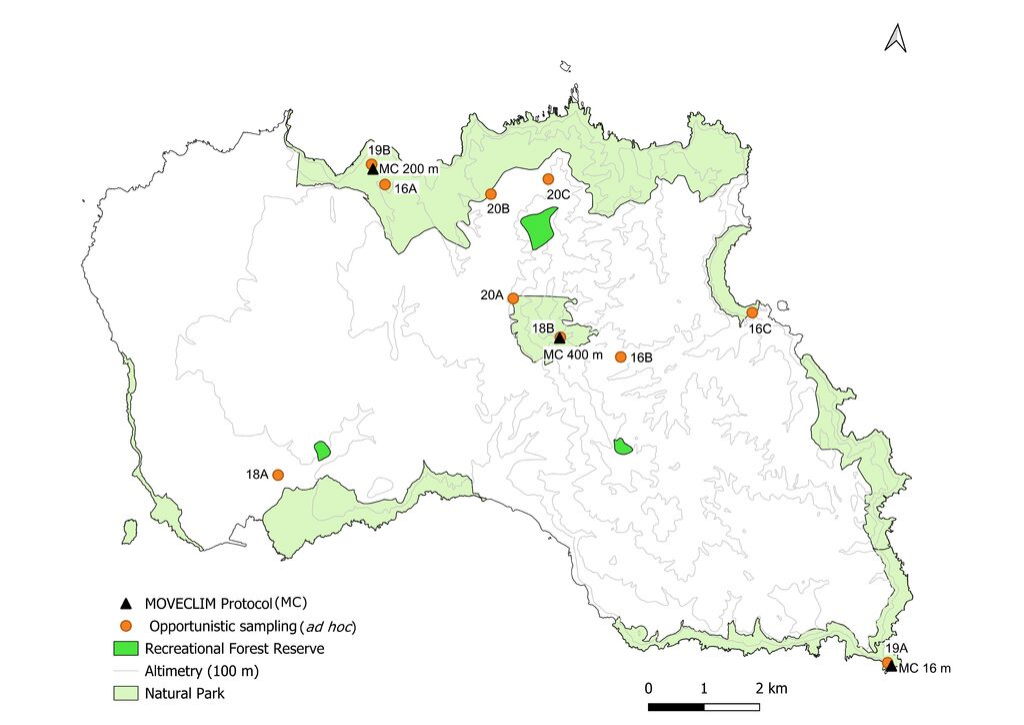
Map of Santa Maria Island indicating the sampling points for the MOVECLIM protocol and opportunistic (*ad hoc*) sampling, including the boundaries of the Natural Park and the Recreational Forest Reserve.

The sampling location and coordinates are listed in Table 1 and Fig. 1.

### Quality control

**Field sampling**: Specimen collection was made by trained bryologists, with careful attention to minimise disturbance and avoid excessive removal of material from natural habitats.

**Storage**: After collection, microplots were placed in paper bags, kept open and separated in a darkened room until fully dried. Once identified, specimens were transferred to labelled herbarium envelopes and deposited in the Herbarium of the University of the Azores (AZU), Section Bryophytes, under the collection name “MOVECLIM – AZORES project: Bryophytes from Santa Maria Island (2019)”.

**Taxonomic identification**: Specimens were identified using the most up-to-date keys and floras, under the supervision of experienced bryologists. Difficult specimens were referred to specialists and extremely small or etiolated material was not identified to species level. Mosses were identified following Smith (2004) and Casas et al. (2006) and liverworts following Paton 1999, Schumacker and Vána 2005, Casas et al. 2009. Additional visual guides and online resources were consulted (e.g. Atherton et al. (2010), Lüth (2019); BBS Field Guide; Bildatlas der Moose Deutschlands). Nomenclature adhered to Gabriel et al. 2010 and Gabriel et al. 2011, incorporating updates available through the PBA (2026).

### Step description

**Obtaining samples**

A research framework was established to improve knowledge of bryophyte flora inhabiting native ecosystems of Santa Maria Island, to document species diversity, distribution patterns and substrate preferences.

MOVECLIM study sites were selected along an altitudinal gradient, giving priority to the best areas of native vegetation, with minimal anthropogenic disturbance. Additional sites were surveyed opportunistically during the prospection to complement the stratified sampling design and maximise habitat coverage. Five of the six substrate types were surveyed, but humus was not found in the survey plots.

Collected specimens were air-dried in a shaded, well-ventilated environment to preserve morphological characters and prevent degradation before identification. In the laboratory, samples were examined under a stereomicroscope and identified to the lowest possible taxonomic level using standard floras and identification keys.

**Data Preparation and Sharing**

A sampling-event dataset, comprising 264 records, was compiled, including detailed metadata on locality, geographic coordinates, elevation, substrate type and vegetation characteristics. An associated occurrence extension dataset was then created, containing 810 records, each corresponding to a bryophyte taxon recorded within the sampled microplots (see Morgado et al. (2026)). Taxonomic nomenclature was standardised according to the most recent references (PBA 2026) to ensure data interoperability and comparability with global biodiversity databases.

## Geographic coverage

### Description

The study was conducted in the Municipality of Vila do Porto, the only municipality of Santa Maria Island, Azores, Portugal. Sampling sites were distributed across a broad spatial extent of the Island, including areas within the Natural Park, the Recreational Forest Reserve, as well as sites located outside protected zones (Fig. 1).

### Coordinates

36.929 and 37.004 Latitude; -25.143 and -25.017 Longitude.

## Taxonomic coverage

### Description

The dataset includes bryophytes from the phyla Anthocerotophyta (hornworts), Bryophyta (mosses) and Marchantiophyta (liverworts).

## Temporal coverage

### Notes

The sampling was performed during 16-20 September 2019.

## Usage licence

### Usage licence

Creative Commons Public Domain Waiver (CC-Zero)

## Data resources

### Data package title

The MOVECLIM – AZORES project: Bryophytes from Santa Maria Island (2019)

### Resource link

https://doi.org/10.15468/a7hvsm

### Number of data sets

2

### Data set 1.

#### Data set name

Event table

#### Data format

Darwin Core Archive

#### Character set

UTF-8

#### Download URL

https://ipt.gbif.pt/ipt/resource?r=moveclim-azo-bry-sma_2019

#### Data format version

1.3

#### Description

The dataset was published on the Global Biodiversity Information Facility (GBIF) platform. The core data file contains 264 event records (eventID). The data and associated resource metadata are available for download through the Portuguese GBIF Portal IPT (Morgado et al. 2026).

**Data set 1.** 

Column label	Column description
eventID	Identifier of the events, unique for the dataset.
parentEventID	An identifier unique for the broader dwc: Event specific to the dataset.
type	Type of the record, as defined by the Dublin Core Standard.
datasetName	Name of the dataset.
samplingProtocol	The sampling protocol used to capture the species.
eventDate	The date-time or interval during which an Event occurred.
day	The day of the month on which the Event occurred.
month	The month in which the Event occurred.
year	The year in which the Event occurred.
habitat	The habitat for an Event.
continent	The name of the continent in which the Location occurs.
country	Country of the sampling site, always Portugal in the dataset.
countryCode	ISO code of the country of the sampling site, always PT in the dataset.
islandGroup	Name of archipelago, always Azores in the dataset.
island	Name of the island, always Santa Maria in the dataset.
municipality	Municipality of the sampling site, always Vila do Porto in the dataset.
locality	The specific description of the place.
locationID	An identifier specific to the dataset.
verbatimElevation	The original description of the elevation (m a.s.l.) of the location.
decimalLatitude	Approximate centre point decimal latitude of the field site in GPS coordinates.
decimalLongitude	Approximate centre point decimal longitude of the field site in GPS coordinates.
geodeticDatum	Standard Global Positioning System coordinate reference for the location of the sample collection points.
coordinateUncertaintyInMetres	Uncertain value of coordinate metrics.
coordinatePrecision	Value in decimal degrees to a precision of six decimal places.
georeferenceSources	Navigation system used to record the location of sample collections.

### Data set 2.

#### Data set name

Occurrence Table

#### Data format

Darwin Core Archive format

#### Character set

UTF-8

#### Download URL

https://ipt.gbif.pt/ipt/resource?r=moveclim-azo-bry-sma_2019

#### Data format version

1.3

#### Description

The dataset was published in the Global Biodiversity Information Facility (GBIF) platform. The core data file contains 810 records (occurrenceID). The data and associated resource metadata are available for download through the Portuguese GBIF Portal IPT (Morgado et al. 2026).

**Data set 2.** 

Column label	Column description
eventID	Identifier of the events, unique for the dataset.
licence	Reference to the licence under which the record is published.
institutionID	The identity of the institution publishing the data.
institutionCode	The code of the institution publishing the data.
collectionID	Identifier of the collection, unique for each specimens are conserved.
collectionCode	The code of the collection where the specimens are conserved.
datasetName	Project reference.
type	Characteristics of the object of study.
basisOfRecord	The nature of the data record.
occurrenceID	Identifier of the record, coded as a global unique identifier.
recordNumber	An identifier given to the Occurrence at the time it was recorded.
recordedBy	A list of names of people, groups or organisations responsible for recording the original Occurrence.
identifiedBy	A list of names of people, who made the identification.
dateIdentified	Date of species identification.
disposition	The current state of a specimen with respect to the collection identified in collectionCode or collectionID.
taxonRank	Lowest taxonomic rank of the record.
kingdom	Kingdom name.
phylum	Phylum name.
class	Class name.
order	Order name.
family	Family name.
genus	Genus name.
specificEpithet	Specific epithet.
infraspecificEpithet	Infraspecific epithet at subspecies level.
scientificNameAuthorship	The authorship information for the scientificName, formatted according to the conventions of the applicable nomenclaturalCode.
scientificName	Complete scientific name including author.
organismQuantity	A number or enumeration value for the quantity of organisms (i, solitary specimen - one or few individuals; p, occasional and less than 5% cover; 1, less than 5% cover of total area; 2, 5% - 25% of total area; 3, 25% - 50% of total area; 4, 50% - 75% of total area; 5, 75% - 100% of total area).
organismQuantityType	Braun-Blanquet Scale.
establishmentMeans	The process of establishment of the species in the location, using a controlled vocabulary: 'Azores endemic', 'European endemic', 'Macaronesian endemic', 'Native', Introduced.
dynamicProperties	A list of additional measurements, facts, characteristics or assertions about the record, including IUCN categories and colonisation status of taxa following the standard notation used for bryophytes.
occurrenceRemarks	Remarks on the material or surface where the biological specimen was collected.

## Additional information

### Data analysis

Species richness, frequency and substrate preferences were recorded for each locality and elevation band. Species were assigned to biogeographical origin categories (Azorean endemic, Macaronesian endemic, European endemic, native or introduced) (PBA 2026) and, where possible, also to an extinction risk category, using IUCN criteria (Endangered, Vulnerable, Near threatened, Least concern, Not applicable or Not evaluated) (Hodgetts et al. 2019) (Suppl. material 2).

### Bryophyte diversity and novelties

The 264 sampling events resulted in a total of 810 specimens, of which the vast majority (98.4%; n = 810) (see Morgado et al. (2026)) were identified to genus, species, subspecies or variety level (Suppl. material 2). Identified specimens represented three phyla: Anthocerotophyta (n = 1), Bryophyta (n = 331) and Marchantiophyta (n = 478). Bryophyta exhibited the highest species richness (S = 45), followed by Marchantiophyta (S = 40) (see Morgado et al. (2026)).

The GIMS systematic sampling protocol (MOVECLIM dataset) yielded the highest number of newly-recorded species for the Island (n = 11), comprising four mosses and seven liverworts. Opportunistic sampling contributed six additional moss species. A further eight newly-recorded species (four mosses and four liverworts) were detected using both sampling approaches.

### Distribution across elevational zones

**Figure 2.**
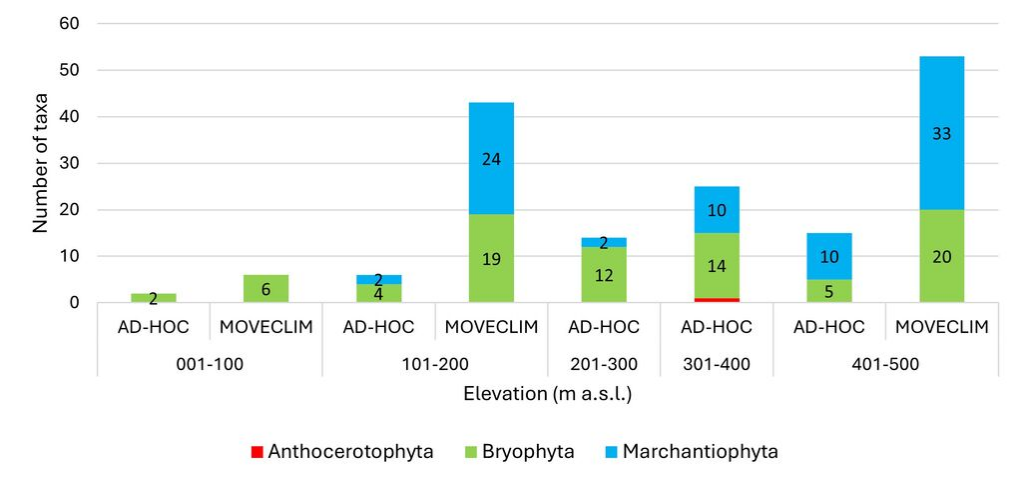
Number of taxa by elevation range (m a.s.l.) on Santa Maria Island (Azores, Portugal).

Mosses and liverworts were collected across all elevation ranges, while a single hornwort specimen was collected at 317 m a.s.l., in a mixed native-exotic woodland. Taxonomic richness increases with elevation, a pattern that is particularly evident in the systematic approach, which includes three elevation levels (16 m, 200 m, 400 m a.s.l.). Intermediate and higher elevations show higher numbers of taxa, while at the lowest elevation range (1–100 m), only a few moss species were recorded, with comparatively low abundance (Fig. 2).

The systematic protocol records a higher number of taxa than the non-systematic approach, which shows lower and more variable values across elevation ranges (Fig. 2). However, as the stratified protocol involved a substantially greater sampling effort than the opportunistic survey, the results are not directly comparable; rather, the two approaches are complementary, as newly-recorded species were detected by both methods.

### Diversity across substrate types

**Figure 3.**
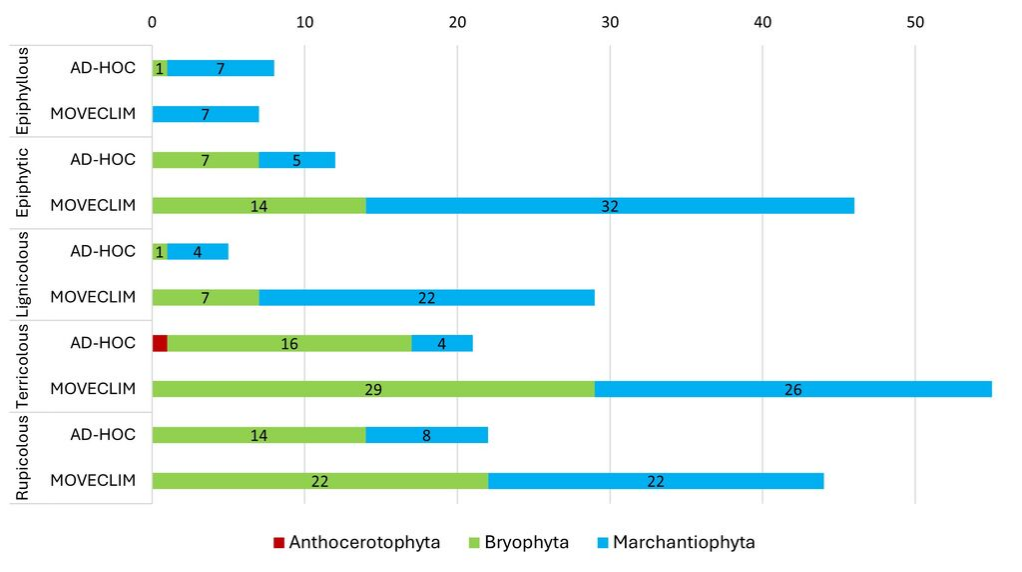
Number of taxa by substrate (rupicolous, terricolous, lignicolous, epiphytic and epiphyllous) on Santa Maria Island (Azores, Portugal).

Bryophyte species were recorded across five substrate types, with soil, bark and rock supporting the highest numbers of bryophyte taxa in both sampling approaches (Fig. 3). Both liverworts and mosses were common on those three substrates, while epiphyllous species were predominantly represented by Marchantiophyta. Anthocerotophyta showed a very restricted distribution, being recorded only once on a terricolous substrate.

Across all substrate categories, the MOVECLIM protocol recorded more taxa than the *ad hoc* survey, reflecting its greater sampling effort.

### Origin and colonisation status

The large majority of the bryophytes collected in this study are classified as native (n = 63); however, there are also endemic elements (13 European endemics; five Macaronesian endemics; two Azorean endemics) and two introduced species (PBA 2026).

The two Azorean endemic *taxa*, Leptoscyphus porphyrius subsp. azoricus and *Rhynchostegiella azorica* are reported for the first time from Santa Maria. Another novelty for the Island is the invasive alien moss *Campylopus introflexus*, a species previously reported from most of the Azorean islands, including São Miguel, Terceira, Pico, São Jorge and Flores (PBA 2026).

### Species of particular interest – protection and management

Seven of the recorded species are currently classified in threatened categories of the IUCN Red List (Hodgetts et al. 2019), including four Vulnerable (VU) and three Endangered (EN) taxa. In addition, eleven species are categorised as Near Threatened (NT), while most of the remaining taxa are listed as Least Concern (n = 65). One species, *Hypnum resupinatum*, has not been evaluated (NE) and the invasive alien moss, *Campylopus introflexus*, is rated as Not Applicable (NA) (Suppl. material 2). The latter species is native to the Southern Hemisphere (South America, South Africa and Australasia and several subantarctic islands) (Hasse 2007), but, in Europe and Macaronesia, it is considered a threat to natural ecosystems (Gama et al. 2017). Its presence is well-known in oceanic regions, particularly in areas influenced by human activities, such as landscapes dominated by plantations of non-native tree species (Sérgio et al. 2018). Whether conservation concern or exotic invasive species, these bryophytes would benefit from dedicated management measures.

### Conclusions

This study improves current knowledge of bryophytes on Santa Maria Island by adding 25 new species (14 mosses and 11 liverworts) and documenting a total of 85 species during a five-day survey. Seven of these species are considered conservation concern according to the IUCN, five liverworts - two of them newly recorded Azorean endemics - and two mosses. In addition, one invasive exotic moss was detected. Overall, this study expands the known distribution of bryophyte species within the Archipelago and underscores the importance of continued monitoring of the islands’ bryophyte flora, natural bioindicators, both for conservation and management of natural areas.

Regarding elevational distribution patterns within the Island, taxonomic richness increases with elevation up to medium-high altitudes. Marchantiophyta become more prominent at higher elevations, whereas Bryophyta occur across the entire altitudinal gradient and Anthocerotophyta remain rare, being recorded only at 317 m. Species were recorded on most substrates studied, with soil supporting the highest number of taxa.

Finally, the stratified and standardised GIMS protocol, within the MOVECLIM project, recorded more taxa than the opportunistic *ad hoc* survey, reflecting its greater sampling effort. However, both methods detected novelties and different species, highlighting the complementarity of the two approaches.

## Supplementary Material

AE64BFE2-C73C-5FF4-BA22-13C3D0E6C1A8 10.3897/BDJ.14.e189834.suppl1 Supplementary material 1 List of publications mentioning bryophytes in Santa Maria Island (Azores) from 1870 to 2023 Data type Table Brief description List of references mentioning the distribution of bryophytes in Santa Maria Island (Azores, Portugal), from 1870 to 2023. Each row includes information on the year of publication, author(s), full reference and type of publication. File: oo_1546933.csv https://binary.pensoft.net/file/1546933 Rosalina Gabriel & Leila N. Morgado

F29E0F3D-EF8C-5E71-8B94-077E1D0A3413 10.3897/BDJ.14.e189834.suppl2 Supplementary material 2 List of bryophyte *taxa* from Santa Maria Island (Azores, Portugal) Data type Table Brief description List of taxa sampled on Santa Maria Island, with their colonisation status categories and IUCN status. File: oo_1546934.csv https://binary.pensoft.net/file/1546934 Leila N. Morgado & Rosalina Gabriel

## Figures and Tables

**Table 1.** Bryophyte sampling code, number of events (Nº), location, elevation (m) and geographic coordinates (Santa Maria Island, Azores).

Code	Events (Nº)	Locality	Elevation (m a.s.l.)	Latitude (N)	Longitude (W)
**STANDARDISED MOVECLIM PROTOCOL**					
MOV_SMA_016_P1	14	Santo Espírito, Maia, Ponta do Castelo.	14	36.928611	-25.017778
MOV_SMA_016_P2	6		17	36.928611	-25.016944
MOV_SMA_200_P1	40	São Pedro, Paisagem Protegida do Barreiro da Faneca e Costa Norte, Ponta do Pinheiro.	193	37.003333	-25.128333
MOV_SMA_200_P2	32		188	37.003889	-25.128611
MOV_SMA_400_P1	55	Santa Bárbara, Pico Alto.	463	36.978056	-25.088333
MOV_SMA_400_P2	56		468	36.978333	-25.088333
**OPPORTUNISTIC SAMPLING**					
SMA_20190916_A	5	São Pedro, Paisagem Protegida do Barreiro da Faneca e Costa Norte, Ponta do Pinheiro.	209	37.000947	-25.125694
SMA_20190916_B	7	Santa Bárbara, Miradouro da Pedra Rija.	373	36.975625	-25.075736
SMA_20190916_C	2	Santa Bárbara, São Lourenço.	79	36.984231	-25.049783
SMA_20190918_A	2	Vila do Porto, Cruzeiro.	88	36.952780	-25.143300
SMA_20190918_B	7	Santa Bárbara, Pico Alto.	464	36.978158	-25.088200
SMA_20190919_A	2	Santo Espírito, Baía da Maia.	31	36.929061	-25.017744
SMA_20190919_B	6	São Pedro, Paisagem Protegida do Barreiro da Faneca e Costa Norte, Ponta do Pinheiro.	185	37.004067	-25.128672
SMA_20190920_A	14	São Pedro, Alto do Nascente.	318	36.983928	-25.098244
SMA_20190920_B	7	São Pedro, Ermida de Nossa Senhora de Fátima.	236	37.000556	-25.104167
SMA_20190920_C	9	São Pedro, matinha da Caldeira.	301	37.003611	-25.092778
